# Deep Learning and High-Resolution Anoscopy: Development of an Interoperable Algorithm for the Detection and Differentiation of Anal Squamous Cell Carcinoma Precursors—A Multicentric Study

**DOI:** 10.3390/cancers16101909

**Published:** 2024-05-17

**Authors:** Miguel Mascarenhas Saraiva, Lucas Spindler, Thiago Manzione, Tiago Ribeiro, Nadia Fathallah, Miguel Martins, Pedro Cardoso, Francisco Mendes, Joana Fernandes, João Ferreira, Guilherme Macedo, Sidney Nadal, Vincent de Parades

**Affiliations:** 1 Department of Gastroenterology, São João University Hospital, Alameda Professor Hernâni Monteiro, 4200-427 Porto, Portugal; tiagofcribeiro@outlook.com (T.R.); miguel.pedro96@gmail.com (M.M.); pedromarilio@gmail.com (P.C.); francisco.cnm@gmail.com (F.M.); guilhermemacedo59@gmail.com (G.M.); 2 WGO Gastroenterology and Hepatology Training Center, 4200-427 Porto, Portugal; 3 Faculty of Medicine, University of Porto, Alameda Professor Hernâni Monteiro, 4200-427 Porto, Portugal; 4 Department of Proctology, GH Paris Saint-Joseph, 185, Rue Raymond Losserand, 75014 Paris, France; lspindler@ghpsj.fr (L.S.); nfathallah@ghpsj.fr (N.F.); vdeparades@ghpsj.fr (V.d.P.); 5 Department of Surgery, Instituto de Infectologia Emílio Ribas, São Paulo 01246-900, Brazil; thiagomanzione@hotmail.com (T.M.); srnadal@terra.com.br (S.N.); 6 Faculty of Engineering, University of Porto, Rua Dr. Roberto Frias, 4200-465 Porto, Portugal; joana.fernandes@digestaid.health (J.F.); j.ferreira@fe.up.pt (J.F.); 7 DigestAID—Artificial Intelligence Development, Rua Alfredo Allen, 4200-135 Porto, Portugal

**Keywords:** high-resolution anoscopy, anal squamous cell carcinoma, high-grade squamous intraepithelial lesion, low-grade squamous intraepithelial lesion

## Abstract

**Simple Summary:**

High-resolution anoscopy (HRA) is crucial for spotting and treating early signs of anal cancer. The researchers created an artificial intelligence (AI) system to analyze HRA images and identify high-grade and low-grade lesions accurately. They trained a computer program with thousands of images, achieving a remarkable accuracy of 94.6%. The AI system proved effective across different examination methods, such as using acetic acid or lugol iodine, and even after treatment. This advancement could improve the early detection of anal cancer precursors, potentially saving lives.

**Abstract:**

High-resolution anoscopy (HRA) plays a central role in the detection and treatment of precursors of anal squamous cell carcinoma (ASCC). Artificial intelligence (AI) algorithms have shown high levels of efficiency in detecting and differentiating HSIL from low-grade squamous intraepithelial lesions (LSIL) in HRA images. Our aim was to develop a deep learning system for the automatic detection and differentiation of HSIL versus LSIL using HRA images from both conventional and digital proctoscopes. A convolutional neural network (CNN) was developed based on 151 HRA exams performed at two volume centers using conventional and digital HRA systems. A total of 57,822 images were included, 28,874 images containing HSIL and 28,948 LSIL. Partial subanalyses were performed to evaluate the performance of the CNN in the subset of images acetic acid and lugol iodine staining and after treatment of the anal canal. The overall accuracy of the CNN in distinguishing HSIL from LSIL during the testing stage was 94.6%. The algorithm had an overall sensitivity and specificity of 93.6% and 95.7%, respectively (AUC 0.97). For staining with acetic acid, HSIL was differentiated from LSIL with an overall accuracy of 96.4%, while for lugol and after therapeutic manipulation, these values were 96.6% and 99.3%, respectively. The introduction of AI algorithms to HRA may enhance the early diagnosis of ASCC precursors, and this system was shown to perform adequately across conventional and digital HRA interfaces.

## 1. Introduction

High-resolution anoscopy (HRA) comprises a diagnostic technique using a colposcope (or a dedicated hardware) for magnification, after the application of acetic acid and lugol to identify anal lesions [1,2]. In the context of anal squamous cell carcinoma (ASCC), HRA plays a central role for the identification of precursor lesions, specifically high-grade intraepithelial lesions (HSIL). The screening of high-risk populations, including men who-have-sex-with-men living with HIV, history of vulvar cancer, and solid-organ transplant recipients, is aimed at detecting these high-risk lesions [3,4,5,6]. Indeed, the identification and treatment of these lesions allows one to prevent the development of ASCC, therefore mitigating the morbidity and mortality associated with ASCC [7,8]. The recently issued International Anal Neoplasia Society (IANS) guidelines reflect the prognostic importance of the detection of ASCC precursors and clarify the populations where screening is advisable and, importantly, the role of HRA following an initial screening with digital rectal examination and anal cytology and/or anal high-risk HPV testing [9].

The performance of HRA is limited by the low number of certified practitioners with experience in this field [9]. The proficient performance of this technique requires extensive training, which limits the widespread application of this technique by experts in the field. Moreover, although the IANS does not clearly define a minimum HRA number to ensure proficiency, the IANS recommends a minimum of 100 HRAs per year as an adequate volume of practice [1]. Indeed, the histological detection of HSIL appears to be dependent on extensive training and has a long learning curve, with increasing slope as more procedures are performed. In a study from 2019, Neukam et al. anticipate that this long learning process could be shortened by the application of artificial intelligence (AI) algorithms [10]. Indeed, diagnostic techniques based on imaging are expected to benefit greatly with the development of deep learning algorithms for their automatic classification. In this regard, convolutional neural networks (CNNs) constitute a deep learning architecture which is particularly designed for image analysis. This type of algorithms has shown great potential for the automatic analysis of medical images across several medical fields [11,12,13,14]. The development of AI algorithms for application to HRA has recently received interest. A pilot study including HRA images from procedures using a digital videoproctoscope has demonstrated promising results, differentiating HSIL from low-grade squamous intraepithelial lesions (LSIL) with a sensitivity of 91%, a specificity of 90%, and an overall accuracy of 90% [15]. Nonetheless, the development of deep learning algorithms for HRA is hampered by the standard use of conventional colposcopes, which limit the generation of large datasets. Thus, to date, no AI-based algorithms were developed using images from HRA exams using standard colposcopes, as is predicted as the standard of practice by the IANS. Moreover, the capability of AI algorithms to be interoperable across distinct technique variations and device models is pivotal to ensure the clinical applicability of the AI systems. Therefore, this multicentric study aims to demonstrate the development of an interoperable AI system for automatic identification of HSIL and its differentiation from LSIL, using a large dataset of HRA images, comprising both exams with a standard colposcope and digital videoproctoscope.

## 2. Materials and Methods

### 2.1. Study Design and Patient Selection

This study includes patients submitted to HRA between 2020 and 2023 at two specialized centers in France (Groupe Hospitalier Paris Saint-Joseph [GHPSJ], Paris, France) and Brazil (Emílio Ribas Infecciology Institute [ERII], São Paulo, Brazil). The exams from the latter center were performed using a conventional colposcope (KLP 200 LED^®^, Kolplast, Bairro da Mina, Briza) while those from the former were performed using a high-resolution videoproctoscope THD^®^ Proctostation HRA Module (THD SpA, Correggio, Italy). At both centers, each procedure was recorded in video format. These videos were stored in “.avi” format and afterwards were segmented into still images using a VLC media player (VideoLAN, Paris, France). The images from both centers were retrospectively reviewed. Images representing the anal transition zone were selected as images of interest for ultimate classification, according to a histological confirmation of HSIL and LSIL.

This study was approved by the institutional review board of Groupe Hospitalier Paris Saint-Joseph (IRB 00012157) and (SPTC 81/2023). This study had a non-interventional design, and all proceedings of this study’s protocol followed the statements of the declaration of Helsinki.

### 2.2. High-Resolution Anoscopy Procedures

For this interoperability study, we developed a dataset including HRA procedures performed both using a conventional colposcope (KLP 200 LED^®^, Kolplast) and a high-resolution videoproctoscopy system (THD^®^ Proctostation HRA Module, THD SpA, Italy). The procedures were performed by four coloproctologists with expertise in HRA (L.S., N.F., T.M. and S.N.). The images were included from patients with histologically proven HSIL or LSIL. This classification was put forward by pathologists at each center with experience in anal pathology and followed the College of American Pathologists protocol [16]. HRA procedures were conducted with the application of a 5% acetic acid solution followed by a lugol iodine solution, if needed. We included images from both categories in distinct settings, specifically previous to any staining, staining with either acetic acid or lugol staining, and during the therapeutic manipulation of the anal canal (e.g., after radiofrequency ablation, laser ablation, infrared coagulation, plasma coagulation or surgical ablation).

### 2.3. Image Processing, Dataset Organization and Development of the Convolutional Neural Network

The main analysis in this study was the capability of the CNN to differentiate between images showing evidence of HSIL vs. LSIL. At this stage, the full dataset (57,822 images) was divided into training (n = 46,163) and testing (n = 11,659) datasets, at a rate of 80% and 20%, respectively.

A secondary analysis was performed to assess the influence of staining and therapeutic intervention on the performance of the CNN. For this stage, four datasets were designed using images with 5% acetic acid staining (n = 27,191), staining with lugol (n = 10,011) and after the therapeutic manipulation of the canal anal (n = 11,047). The latter subset of images included frames collected during in-office therapeutic procedures, at different stages of completion, which were classified by experts as showing areas compatible residual lesions, within areas of previously defined HSIL. For each of these subsets, images were divided into training and testing datasets at a similar ratio used in the main analysis.

For the learning of the CNN, a circular region of interest (ROI) was identified in white for all images. Parameter optimization of the HoughCircles filter from OpenCV to one circular ROI was used for each frame [17]. Subsequently, masks, contours, and crop functions were employed to place the extracted ROI in the center of a black image, mirroring the original.

The deep learning model was generated using *Resnet* as its fundamental structure, with weights trained on ImageNet. We facilitated knowledge transfer to our dataset by preserving the existing model architecture. The final fully connected layers were excised, and in their place, new fully connected layers tailored were appended to accommodate the specific number of classes employed for HRA image classification. Two blocks were used, each with a fully connected layer, followed by a dropout layer with a drop rate of 0.3. Subsequently, we included a dense layer with a size defined as the number of categories to classify. By trial and error, we defined a learning rate of 0.0001, a batch size of 32, and a number of epochs of 10. Pytorch was used to run the model after preparation of the data using *FFMPEG*, *Pandas,* and *Pillow* libraries. The analyses were performed with a computer equipped with a 2.1 GHz Intel^®^ Xeon^®^ Gold 6130 processor (Intel, Santa Clara, CA, USA) and a single NVIDIA^®^ RTX™ A6000 graphic processing unit (NVIDIA Corporate, Santa Clara, CA, USA).

### 2.4. Model Performance and Statistical Analysis

At each experimental stages, the output provided by CNN was compared to the gold-standard histology (HSIL vs. LSIL). After training and hyperparameter optimization, the network computed the probability for each category for every image. The performance metrics encompass sensitivity, specificity, positive, and negative predictive values (PPV and NPV, respectively), and accuracy. Furthermore, the discriminative efficacy of each model was assessed through the analysis of receiver operating characteristic (ROC) curves. Additionally, the computational efficiency of the Convolutional Neural Network (CNN) was ascertained by calculating the processing time required for the CNN to generate output for the entire set of images in the validation image dataset. Sci-Kit learn version 0.22.2 [18] was used for statistical analysis.

## 3. Results

A total of 151 HRA exams were performed in 137 patients from both centers. From this group, 92 patients were included from GHPSJ (n = 106 exams) and 45 patients from ERII (n = 45 exams). A total of 57,822 images were extracted and used for building and developing the algorithm, from which 32,497 originated after examinations with a high-resolution videoproctoscope, and the remaining with a conventional colposcope (n = 25,325). Ultimately, from the total pool of images, 28,874 showed lesions with histological evidence of HSIL and 28,948 showed LSIL.

**Figure 1:**
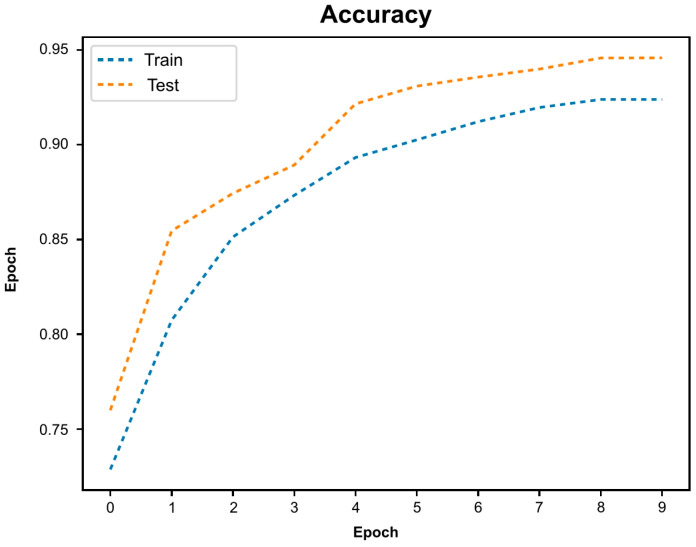
Evolution of the algorithm’s accuracy during training and testing stages.

**Figure 2:**
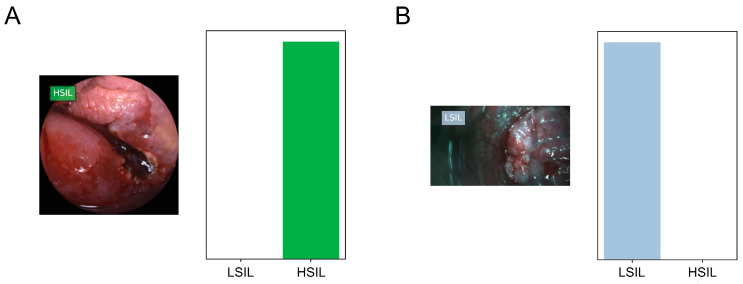
Output obtained after running the convolutional. (**A**)—High-resolution videoproctoscope; (**B**)—conventional colposcope. HSIL—high-grade squamous intraepithelial lesion; LSIL—low-grade squamous intraepithelial lesion.

Figure 1 represents the evolution the accuracy of the algorithm during training and validation, demonstrating increasing accuracy with the exposure to a higher volume of data. During each stage, the CNN would predict the probability of any given frame belonging to each of the classification categories (i.e., HSIL or LSIL). The category with the highest probability was outputted as the network’s prediction (Figure 2).

For a first analysis, the performance for the automatic detection and differentiation of HSIL versus LSIL. For this purpose, the full dataset was divided according to a distribution of 80% (n = 46,798, from which 23,369 showed HSIL) for training and 20% (n = 11,700, from which 5843 showed HSIL) for testing of the model. At this first stage, the model achieved a sensitivity of 93.6%, specificity of 95.7%, PPV of 95.6%, NPV of 93.7%, and an overall accuracy of 94.6% (AUC 0.97).

In the second experiment, the subsets of images were organized to assess the performance of the algorithm according to different procedure stages during HRA procedures. These subsets were constituted by HRA images after acetic acid staining (n = 27,191), after lugol iodine staining (n = 10,011) and during therapeutic interventions (n = 11,047). For each subset of images, training and validation sets were organized using a similar distribution as previously referred to for the first experiment.

**Figure 3:**
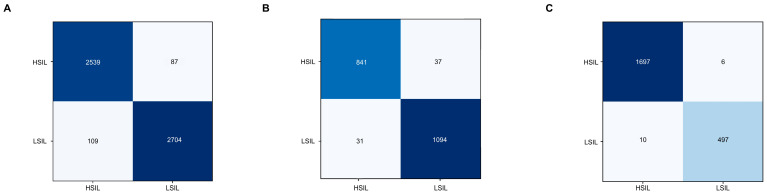
Confusion matrices for each subanalysis group. (**A**)—acetic acid; (**B**)—lugol iodine; (**C**)—post-manipulation.

The confusion matrices for the testing dataset for each subanalysis group are shown in Figure 3. In the testing dataset, when evaluating frames showing the squamocolumnar area stained with 5% acetic acid, the CNN reached a sensitivity of 96.7%, a specificity of 96.1%, a PPV of 95.9%, a NPV of 96.9%, and an overall accuracy of 96.4%. The AUC for the differentiation between both categories was 0.98. For images stained with lugol iodine, the CNN differentiated HSIL from LSIL with a sensitivity of 95.8%, a specificity of 97.2%, a PPV and NPV of 96.4% and 96.7%, respectively, and an accuracy 96.6%. Within this subset of images, the algorithm achieved an AUC of 0.99. Finally, in the setting after therapeutic procedures during HRA exams, the algorithm detected and differentiated HSIL from LSIL with a sensitivity of 99.6%, a specificity of 98.0%, a PPV and NPV of 99.4% and 98.8%, respectively, and an overall accuracy of 99.3% (AUC 1.00).

## 4. Discussion

The increasing prevalence in the description of new deep learning algorithms in the field of gastroenterology opens a new window into the optimization of optical diagnosis during endoscopic procedures [19,20,21]. While these algorithms are steadily receiving regulatory approval and entering clinical practice in the case of systems applied to conventional gastrointestinal endoscopy, the development of AI algorithms for anorectal diagnostic methods remain scarcely explored [15]. Moreover, there are significant challenges hampering the integration of AI algorithms in clinical practice. One of the most significant challenges concerns the lack of interoperability between different diagnostic systems, therefore restricting the access of patients to medical care and clinical information, as well as limiting the access to high-quality real-world data for clinical and translational investigation [22,23].

To our knowledge, this is the first study to develop a deep learning algorithm which is capable of working simultaneously on distinct HRA platforms, with significant procedural and system requirements, therefore addressing the issue of interoperability. This system extends the scope of the system that has been previously described by our group [15]. Indeed, this extension is particularly relevant in the field of HRA, as most Proctology centers perform HRA using conventional colposcopes. Thus, the redefinition of the algorithm to accommodate HRA performed with conventional colposcopes will expand the reach of this technology, which has been shown, in this study, to have an adequate performance when considering both HRA systems. This algorithm was developed by complying with methods that will allow its communication with multiple devices. This, in line with the FAIR principles, which were issued in 2016 to serve as a guide for data management and stewardship [24]. These principles indicate that data should be made findable, accessible, interoperable and reusable. Indeed, in this study efforts were instituted to ensure the compliance with these principles. For example, we standardized the collection of data using single-entry anonymized records, which are easily found within this study’s database. This also contributes to ensuring easy access to patients’ data by the study investigators, simultaneously safeguarding the privacy of data. For the first time, our group addressed the interoperability issue by developing an AI model capable of working across several systems. The topic of interoperability has derived from data management and several studies have advocated the importance of its application to the development of AI algorithms in medicine [22,25].

The identification of HSIL is of particular importance as its presence implies a greater risk of the development of ASCC. This risk is particularly higher in vulnerable segments of the population, most significantly people living with HIV [26]. The pivotal ANCHOR study has provided robust evidence on the outcome benefit of identifying and treating patients with this ASCC precursor lesions. This study has shown that, in a population of adults living with HIV, those for whom HSIL had been identified and treated had a 57% lower risk of progressing to ASCC [8]. The dual role of HRA, providing detection and the possibility of treatment of precursor lesions are the main justifications for the reduction in the risk of ASCC. Nonetheless, the technique requires extensive training and has limited availability [27,28]. Indeed, the IANS acknowledges that these limitations may hamper the applicability of its practice recommendations [9].

The IANS has developed practice standards for the practice of HRA [1]. These clinical practice guidelines contemplate the performance of the technique using conventional colposcopes. Nevertheless, despite the technical advantages provided by the use of high-resolution videoproctoscopes, these systems have not received such endorsement. This study extends the findings of previous studies by our group where a system design to be applied to a single high-resolution videoproctoscope system was designed. That deep learning algorithm achieved an overall accuracy for the detection of HSIL and its distinction from LSIL [15]. In the present study, our group has modified the algorithm to also be applicable to standard HRA systems. Indeed, this system has been shown to have a high-performance level, with a sensitivity and specificity of 94% and 96%, respectively, and an overall accuracy of 95%. This incremental step is extremely relevant as it represents the extension of the algorithm to an IANS-endorsed diagnostic technique. Moreover, in this multicenter study, we provide an extension of the system to the standard HRA technique, therefore allowing to demonstrate the performance of these innovative algorithms applied to a widely used technique. This preliminary step is required for the future application of this software for all HRA settings. Finally, integrating images from both types of HRA system, the algorithm retained an adequate discriminating capability between HSIL and LSIL, across the different subanalyses: staining with acetic acid, lugol iodine and during therapeutic interventions. From a technical perspective, the analysis of the subset of images during therapeutic interventions for the treatment of HSIL opens the perspective that the real-time application of these algorithms may help to identify areas where residual lesion is more probable. Moreover, besides its potential in facilitating the diagnosis, this type of algorithm may contribute to increased HRA availability and potentiate the learning of typical patterns, which is particularly relevant in low-volume settings [29]. Finally, the increase in diagnostic capacity should be accompanied by additional efforts in engaging with this group of patients, so that more effective screening is followed by the adequate management of ASCC precursors [30].

Despite its merits, this study has some limitations. First, despite its multicentric matrix, this study has a retrospective design. This study has the ultimate goal of translating the results of the newly developed algorithm encompassing in its dataset images from different HRA systems. Despite this significant methodological leap, this study is not intended to evaluate the clinical impact of this technology. Second, while we advocate that the algorithm will be most helpful for real-time assistance during HRA exams, and that the ambition will be to increase the yield of HRA-guided biopsies, these analyses described in this study area were based on the evaluation of imagens and not during real-time exams. Finally, comparing the performance of the algorithm independently for imagens from conventional colposcope versus images from high-resolution videoproctoctopes would be helpful to further evaluate the model.

High-resolution anoscopy is expected to benefit greatly from the integration of AI technologies. AI holds significant potential to enhance the accuracy, efficiency, and accessibility in the screening for ASCC precursors. Through advanced image analysis algorithms, AI can assist in the detection and characterization of lesions with greater precision than traditional methods alone. These AI models, trained on the vast datasets of HRA images, have the potential to recognize the subtle patterns indicative of ASCC precursors. This could lead to earlier detection and intervention, ultimately improving patient outcomes and reducing healthcare costs.

In this study, a deep learning algorithm was developed to detect HSIL and differentiate this ASCC precursor from LSIL. The system showed high-performance levels, which were sustained across different staining protocols and after therapeutic procedures. Moreover, our system was interoperable across different HRA systems, both using a conventional colposcope and a high-resolution videoproctoscope. The interoperability is crucial for effective integration into clinical practice.

## 5. Conclusions

The application of AI to HRA can aid healthcare providers in interpreting HRA findings, offering real-time guidance and increasing diagnostic confidence. Overall, the integration of AI into HRA holds promise for revolutionizing anal cancer screening and management, paving the way for more personalized and effective patient care.

## Data Availability

Non-identifiable data will be made available upon reasonable request.
