# Correction: Saraiva et al. Deep Learning and High-Resolution Anoscopy: Development of an Interoperable Algorithm for the Detection and Differentiation of Anal Squamous Cell Carcinoma Precursors—A Multicentric Study. *Cancers* 2024, *16*, 1909

**DOI:** 10.3390/cancers17223698

**Published:** 2025-11-19

**Authors:** Miguel Mascarenhas Saraiva, Lucas Spindler, Thiago Manzione, Tiago Ribeiro, Nadia Fathallah, Miguel Martins, Pedro Cardoso, Francisco Mendes, Joana Fernandes, João Ferreira, Guilherme Macedo, Sidney Nadal, Vincent de Parades

**Affiliations:** 1 Department of Gastroenterology, São João University Hospital, Alameda Professor Hernâni Monteiro, 4200-427 Porto, Portugal; 2 WGO Gastroenterology and Hepatology Training Center, 4200-427 Porto, Portugal; 3 Faculty of Medicine, University of Porto, Alameda Professor Hernâni Monteiro, 4200-427 Porto, Portugal; 4 Department of Proctology, GH Paris Saint-Joseph, 185, Rue Raymond Losserand, 75014 Paris, France; 5 Department of Surgery, Instituto de Infectologia Emílio Ribas, São Paulo 01246-900, Brazil; 6 Faculty of Engineering, University of Porto, Rua Dr. Roberto Frias, 4200-465 Porto, Portugal; 7 DigestAID—Artificial Intelligence Development, Rua Alfredo Allen, 4200-135 Porto, Portugal

## Text Correction

There were errors in the original publication [1] in the Materials and Methods section, subsection Study Design and Patient Selection, paragraph 1, as well as subsection High-Resolution Anoscopy Procedures. It was stated that high-resolution anoscopy exams were performed with high-resolution videoproctoscope THD^®^ HRStation, when they were performed with THD^®^ Proctostation HRA Module. The authors state that the scientific conclusions are unaffected. This correction was approved by the Academic Editor. The original publication has also been updated.

A correction has been made to Materials and Methods, Study Design and Patient Selection, Paragraph 1.

The corrected paragraph appears below:

This study includes patients submitted to HRA between 2020 and 2023 at two specialized centers in France (Groupe Hospitalier Paris Saint-Joseph [GHPSJ], Paris, France) and Brazil (Emílio Ribas Infecciology Institute [ERII], São Paulo, Brazil). The exams from the latter center were performed using a conventional colposcope (KLP 200 LED^®^, Kolplast, Bairro da Mina, Briza) while those from the former were performed using a high-resolution videoproctoscope THD^®^ Proctostation HRA Module (THD SpA, Correggio, Italy). At both centers, each procedure was recorded in video format. These videos were stored in “.avi” format and afterwards were segmented into still images using a VLC media player (VideoLAN, Paris, France). The images from both centers were retrospectively reviewed. Images representing the anal transition zone were selected as images of interest for ultimate classification, according to a histological confirmation of HSIL and LSIL.

A correction has been made to High-Resolution Anoscopy Procedures, Paragraph 1.

The corrected paragraph appears below:

For this interoperability study, we developed a dataset including HRA procedures performed both using a conventional colposcope (KLP 200 LED^®^, Kolplast) and a high-resolution videoproctoscopy system (THD^®^ Proctostation HRA Module, THD SpA, Italy). The procedures were performed by four coloproctologists with expertise in HRA (L.S., N.F., T.M. and S.N.). The images were included from patients with histologically proven HSIL or LSIL. This classification was put forward by pathologists at each center with experience in anal pathology and followed the College of American Pathologists protocol [16]. HRA procedures were conducted with the application of a 5% acetic acid solution followed by a lugol iodine solution, if needed. We included images from both categories in distinct settings, specifically previous to any staining, staining with either acetic acid or lugol staining, and during the therapeutic manipulation of the anal canal (e.g., after radiofrequency ablation, laser ablation, infrared coagulation, plasma coagulation or surgical ablation).
